# A functional unbalance of TRPM8 and Kv1 channels underlies orofacial cold allodynia induced by peripheral nerve damage

**DOI:** 10.3389/fphar.2024.1484387

**Published:** 2024-12-05

**Authors:** Ricardo Piña, Gonzalo Ugarte, Camilo Guevara, Richard Pino, Katherine Valdebenito, Sofía Romero, Ana Gómez del Campo, Víctor Hugo Cornejo, María Pertusa, Rodolfo Madrid

**Affiliations:** ^1^ Departamento de Biología, Facultad de Química y Biología, Universidad de Santiago de Chile, Santiago, Chile; ^2^ Departamento de Biología, Facultad de Ciencias Básicas, Universidad Metropolitana de Ciencias de la Educación, Santiago, Chile; ^3^ Millennium Nucleus of Ion Channel-Associated Diseases - MiNICAD, Santiago, Chile; ^4^ Millennium Nucleus for the Study of Pain - MiNuSPain, Santiago, Chile; ^5^ Facultad de Ciencias Biológicas, Pontificia Universidad Católica de Chile, Santiago, Chile

**Keywords:** primary sensory neurons, thermotransduction, infraorbital nerve, I_KD_, AAV vectors, 4-AP, PBMC

## Abstract

Cold allodynia is a debilitating symptom of orofacial neuropathic pain resulting from trigeminal nerve damage. The molecular and neural bases of this sensory alteration are still poorly understood. Here, using chronic constriction injury (CCI) of the infraorbital nerve (IoN) (IoN-CCI) in mice, combined with behavioral analysis, Ca^2+^ imaging and patch-clamp recordings of retrogradely labeled IoN neurons in culture, immunohistochemistry, and adeno-associated viral (AAV) vector-based delivery *in vivo*, we explored the mechanisms underlying the altered orofacial cold sensitivity resulting from axonal damage in this trigeminal branch. We found that cold allodynia induced by IoN-CCI is linked to an increase in the proportion of cold-sensitive neurons (CSNs) contributing to this branch and a shift in their thermal thresholds to higher temperatures. These changes are correlated to a reduction of the Kv1.1-1.2-dependent brake potassium current I_KD_ in IoN CSNs and a rise in the percentage of trigeminal neurons expressing TRPM8. The analysis of the electrophysiological properties of CSNs contributing to the IoN suggests that painful cold hypersensitivity involves the recruitment of silent nociceptive afferents that become sensitive to mild cold in response to nerve damage. Notably, pharmacological suppression of TRPM8 channels and AAV-based transduction of trigeminal neurons with the Kv1.1 channel *in vivo* effectively reverted the nociceptive phenotype in injured animals. Altogether, our results unveil a crucial role of TRPM8 and Kv1 channels in orofacial cold allodynia, suggesting that both the specific TRPM8-blocking and the AAV-driven expression of potassium channels underlying I_KD_ in trigeminal neurons can be effective tools to revert this damage-triggered sensory alteration.

## 1 Introduction

Painful hypersensitivity to innocuous cold, or cold allodynia, is one of the debilitating symptoms of orofacial neuropathic pain resulting from trigeminal nerve damage, whose molecular and neural bases are not entirely clarified. Under physiological conditions, cold thermoreceptors and cold-sensitive nociceptors are the primary somatosensory neurons responsible for the detection of low temperatures. Cold transduction and cold-induced firing in these neurons occur by the concerted action of several classes of transduction and voltage-gated ion channels, that functionally coexist to give shape to the cold-induced receptor potential and subsequent action potential firing in response to temperature drops (McKemy, 2013; 2018; Vriens et al., 2014; González et al., 2015; Buijs and McNaughton, 2020; Lewis and Griffith, 2022). Among them, the cold-activated Transient Receptor Potential Melastatin 8 channel (TRPM8) has emerged as the main molecular entity responsible for cold sensitivity in primary sensory neurons (McKemy et al., 2002; Peier et al., 2002), whose depolarizing effect is counterbalanced by the brake potassium current I_KD_ dependent on Voltage-gated Potassium channels, family 1, members 1 and 2 (Kv1.1 and Kv1.2) (Viana et al., 2002; Madrid et al., 2009).

We have previously shown that chronic constriction injury of the sciatic nerve induces a functional reduction of I_KD_, shifting the mean cold threshold of CSNs expressing TRPM8 to higher temperatures and, more importantly, transforming a subpopulation of TRPM8(+) nociceptive fibers in neurons responding to innocuous cold, explaining the cold allodynia observed in these animals (González et al., 2017). In oxaliplatin-induced neuropathy, partial sciatic nerve ligation, and ciguatera poisoning models, it also has been suggested that cold allodynia emerges as the result of unmasking silent cold-sensing neurons connected to nociceptive routes, which occurs as a consequence of the downregulation of Kv1 channels in primary sensory neurons expressing Nav1.8 and CGRPα (MacDonald et al., 2021). In contrast, the peripheral injury of corneal nerve fibers causes an increase in cold sensitivity by functional potentiation of TRPM8 activity in trigeminal cold thermoreceptors innervating the ocular surface, without changes in the I_KD_ (Piña et al., 2019). In orofacial territories, several reports have shed light on the role of some thermo-TRP channels and other transduction and voltage-gated channels in the molecular mechanisms underlying pathological pain. Among them, TRPM8 (Rossi et al., 2012; Zuo et al., 2013; Gualdani et al., 2021), TRPA1 and TRPV1 (Rossi et al., 2012; Honda et al., 2014; Urata et al., 2015; Hargreaves and Ruparel, 2016; Trevisan et al., 2016), Kv7.2-7.3 (Abd-Elsayed et al., 2015; Ling et al., 2017; 2018), BKCa (Liu et al., 2015), NMDAR (Liu et al., 2022), Kv1.4 (Yang et al., 2022), and Nav1.7 channels (Loya-Lopez et al., 2024), have been related to the development and maintenance of disabling orofacial pain in response to nerve injury in different animal models, mainly related to altered mechanical pain sensitivity. Nevertheless, despite the advances in the study of damage-triggered painful cold hypersensitivity in different somatosensory territories and pathologies (reviewed by (Yin et al., 2015; Lolignier et al., 2016; Alles and Smith, 2018; Viana and Voets, 2019; MacDonald et al., 2020; Finnerup et al., 2021; Luo et al., 2021)), the specific role of the key counteracting cold-sensitive TRP channel TRPM8 and voltage-gated Kv1 channels underlying the brake current I_KD_, and the subpopulations of primary afferents involved in signaling orofacial cold allodynia remain to be determined.

Here, we have explored the thermal sensitivity, excitability, and neural phenotype of trigeminal neurons involved in pathological cold sensitivity induced by chronic constriction of the infraorbital branch of the trigeminal nerve in mice. Using this form of peripheral nerve damage to induce painful cold hypersensitivity in the vibrissal pad region, we studied the mechanisms underlying the cold-evoked painful response in this specific territory. Our results suggest that cold allodynia in orofacial neuropathic pain is due to a functional unbalance of TRPM8 and the I_KD_ current in IoN neurons. This unbalance shifts the thermal sensitivity of cold thermoreceptors to higher temperatures and recruits former silent cold-insensitive neurons signaling pain. Thus, our findings provide evidence of the neural and molecular mechanisms that underpin this sensory alteration induced by chronic damage of the trigeminal sensory fibers innervating orofacial territories.

## 2 Materials and methods

### 2.1 Animals

This study was performed using young adult (P21-P40) male and female C57BL/6 mice. Animals were housed in a maximum of four per cage in a 12-h light/dark cycle with food and water *ad libitum* and euthanized with CO_2_. All experiments were conducted according to the bioethical guidelines of the *Agencia Nacional de Investigación y Desarrollo de Chile (ANID)* and have been approved by the Bioethical Committee of the Universidad de Santiago de Chile (Protocol Reference Number 259).

### 2.2 Model of orofacial cold allodynia induced by peripheral nerve damage

We used chronic constriction injury of the infraorbital branch of the trigeminal nerve (IoN-CCI) as a model of axonal damage manifesting orofacial cold allodynia in mice as in Constandil et al. (2012). In brief, ligation was performed on the right IoN of each animal anesthetized with ketamine (80 mg/kg) and xylazine (10 mg/kg) under direct visual control using a stereomicroscope. During surgery, mice were gently immobilized on the operating table and kept warm using a heat blanket at 37°C until recovery. The maxillary branch of the trigeminal nerve was exposed by an incision ∼4 mm long in the gingivo-buccal mucosa beginning just cranial to the first molar, and gently freed from surrounding muscles and connective tissue. The lateral part of the branch was ligated with a single 8–0 chromic gut, and the incision was closed. For sham controls, the infraorbital branch was exposed, and the silk was passed through but not tied, carefully avoiding stretching the nerve or damaging the epineurium.

### 2.3 Retrograde labeling of IoN neurons

Trigeminal ganglia (TG) neurons contributing to the IoN and innervating the vibrissal pad skin were retrogradely labeled using FM1-43 fluorescent dye (T35356, Thermo-Fisher Scientific, Waltham, MA, United States of America) on anesthetized animals of both groups, 7 days after the surgical procedure. Mice were immobilized, and the fluorescent marker was applied by intradermal injection of 5 µL of a 3 mM stock in saline solution using a 30-gauge needle coupled to a Hamilton syringe. After FM1-43 application, mice were left with food and water *ad libitum* for 3 days before the preparation of neuronal cultures for electrophysiological and Ca^2+^ imaging experiments, allowing retrograde transportation of the fluorescent marker to the soma of IoN neurons.

### 2.4 Acetone evaporative cooling assay

Cold sensitivity was evaluated using the acetone evaporation assay. Mice were habituated to the behavior room for at least 3 h before testing and to the experimental chambers for at least 20 min before the assay takes place. Experiments were performed during the light period. The same investigators carried out the scoring in all the behavioral tests, which were performed blindly on the type of operation. For acetone-evoked evaporative cooling, mice were placed in round acrylic chambers. A drop (∼10 µL) of acetone (90%) was topically applied to the vibrissal region using a customized blunt needle attached to a microsyringe. Special care was taken to avoid acetone leakage towards the ocular region or the nose. The nociceptive behavior, evaluated as asymmetric orofacial grooming (rubbing and scratching on the vibrissal pad region executed with the ipsilateral fore or hind paw), and the number of the following nocifensive events were monitored during the subsequent 60 s at 0 (basal), 1, 3, 5, 7, and 10 days after nerve injury unless otherwise is indicated. In AVV-based transduction experiments *in vivo*, monitoring was extended until day 33. Nocifensive responses were observed during the first minute after acetone application, and measurements were repeated three times with a 10-min interval to obtain the mean value of the time spent for each mouse in this behavior. Solutions, either 4-aminopyridine (4-AP) or vehicle (saline solution), were injected into the vibrissal region in a volume of 5 µL using a 30-gauge needle coupled to a Hamilton syringe, and orofacial acetone tests were performed 10 min after local injection of the drug or vehicle. For the systemic application of (S)-1-Phenylethyl (2-aminoethyl) (4-(benzyloxy)-3-methoxybenzyl) carbamate (PBMC) (10 mg/kg), solution of either drug or vehicle was injected i.p. as in (Knowlton et al., 2011), and acetone tests were performed 40 min after injection of the drug or vehicle.

### 2.5 AAV-based transduction

All AAV vectors used in this study were manufactured by Vector Biolabs (Malvern, PA, United States of America). AAV5 vectors designed to introduce cDNA of the mKv1.1 channel contained the fluorescent reporter eGFP. Control experiments were conducted using vectors expressing the reporter. For *in vitro* experiments, 24 h after trigeminal neurons were seeded in Poly-L-lysine-coated glass coverslips in 24-well plates, 1.5 × 10^10^ gc of AAV5 vectors were added to the medium. For stereotaxic injections of the trigeminal ganglia *in vivo*, mice were anesthetized, placed into a stereotaxic frame with nose and ear bars, and kept warm using a heat blanket at 37°C until recovery. The vector was delivered in 1 µL of AAV stock of 10^13^ gc/mL using a 5 µL Hamilton syringe with a 32G-4 needle, controlled by a UMP3 microcontroller (WPI, Hertfordshire, UK). Unilateral AAV delivery was administered by injecting the TG at the coordinates: 4.3 mm rostral to the lambda, 1.5 mm lateral to the lambda, and 6.24 mm ventral to the lambda. Injections were conducted at 0.25 μL/min, and the needle was left in place for three additional minutes to allow particle absorption before slow withdrawal. AAV vectors were delivered 1 week after IoN-CCI, when the injured animals reached a stable near-maximum allodynic phenotype.

### 2.6 Cell culture

Pairs of sham and IoN-CCI mice were euthanized by CO_2_ inhalation. TG neurons were cultured as in Piña et al. (2019). In brief, trigeminal ganglion ipsilateral to the nerve injury (or sham surgery) was removed and incubated in an enzymatic mixture including collagenase type XI (650 UI/mL; C7657, Sigma-Aldrich, St. Louis, United States of America) and dispase (5 UI/mL; 17105-041 GIBCO-Thermo Fisher Scientific, Waltham, MA, United States of America), in INC-mix solution (in mM: NaCl 155, K_2_HPO_4_ 1.5, HEPES 10, Glucose 5; pH 7.4), during 40 min at 37°C in 5% CO_2_. TG were then mechanically dissociated using a polished Pasteur pipette, and neurons were plated on poly-L-lysine-coated 6 mm #0 glass coverslips (Menzel-Gläser, Braunschweig, Germany), maintained in MEM media (Earle’s salts, 111095080, GIBCO-Thermo Fisher Scientific, Waltham, MA, United States of America) supplemented with MEM-vit (11120052, GIBCO-Thermo Fisher Scientific, Waltham, MA, United States of America), 10% FBS (SH30910.03, Hyclone, General Electric Healthcare Life Science, UT, United States of America), 200 μg/mL streptomycin, 125 μg/mL penicillin (15140-122, GIBCO-Thermo Fisher Scientific, Waltham, MA, United States of America), and used within 8 to 16 h after plating for [Ca^2+^]_i_ imaging experiments and patch-clamp recordings. Ca^2+^ imaging and patch-clamp experiments were performed in both conditions (sham and IoN-CCI) and tested simultaneously, always 10 days after surgery.

### 2.7 Ca^2+^ imaging

For ratiometric Ca^2+^ imaging experiments, TG neurons were incubated with 5 µM Fura-2 AM (F1221, Invitrogen-Thermo Fisher Scientific, Waltham, MA, United States of America) dissolved in extracellular standard solution for trigeminal neurons (see below) supplemented with 0.02% pluronic acid (P6867, Invitrogen-Thermo Fisher Scientific, Waltham, MA, United States of America) for 50 min at 37°C in darkness. Ratiometric fluorescence measurements were made with an inverted Nikon Ti microscope fitted with a 12-bit cooled ORCA C8484-03G02 CCD camera (Hamamatsu, Hamamatsu City, Japan). Fura-2 was excited at 340 nm and 380 nm at 0.5 Hz with a Polychrome V monochromator (Till Photonics, Thermo-Fisher, Waltham, MA, United States of America), with exposures no longer than 40 ms long, and the emitted fluorescence was filtered with a 510 nm long-pass filter. Fluorescence ratios (at 0.5 Hz) were displayed online with HCImage v2 software (Hamamatsu, Hamamatsu City, Japan). Bath temperature (see below for details) was sampled simultaneously using a Physitemp BAT-12 microprobe thermometer (Physitemp Instruments, Clifton, NJ, United States of America) supplemented with an IT-18 T-thermocouple, using Clampex 10 software (Molecular Devices, Sunnyvale, CA, United States of America) and digitized with an Axon Digidata 1440A AD converter (Molecular Devices, Sunnyvale, CA, United States of America).

Threshold temperature values for [Ca^2+^]_i_ elevation in TG neurons were estimated as described in Madrid et al. (2006); 2009, by linearly interpolating the temperature at the midpoint between the last baseline point and the first point at which a rise in [Ca^2+^]_i_ deviated by at least four times the standard deviation of the baseline. To quantify the percentage of CSNs in both groups of mice, a solution containing elevated K^+^ (30 mM KCl) was perfused at the end of the protocol to determine the viability of the neurons in the entire field. The percentage of cells responding to this depolarizing stimulus is ∼60–70%. Only neurons showing a [Ca^2+^]_i_ increase in response to high extracellular K^+^ solution were included in the analysis of the percentages of IoN CSNs in culture.

### 2.8 Electrophysiology

In patch-clamp experiments, whole-cell current and voltage signals were recorded using an Axopatch 200B amplifier (Molecular Devices, Sunnyvale, CA, United States of America), and the temperature was monitored simultaneously in voltage- or current-clamp recordings. The extracellular standard solution for trigeminal neurons contained (in mM): 140 NaCl, 3 KCl, 1.3 MgCl_2_, 2.4 CaCl_2_, 10 HEPES, 10 glucose (298 mOsm/kg pH 7.4, adjusted with NaOH). Standard patch-clamp pipettes (4–5 MΩ resistance) were made using GC150F-7.5 glass capillaries (Harvard Apparatus, Holliston, MA, United States of America) and filled with intracellular solution containing (in mM): 105 K-gluconate, 35 KCl, 8.8 NaCl, 10 HEPES, 0.5 EGTA, 4 MgATP, 0.4 NaGTP, (300 mOsm/kg and pH 7.4, adjusted with KOH). Stimulus delivery and data acquisition were performed using pClamp 10 software (Molecular Devices, Sunnyvale, CA, United States of America). Before electrophysiological recordings in IoN CSNs, the temperature threshold was determined using Ca^2+^ imaging.

### 2.9 Immunostaining

Anesthetized animals were perfused via the left ventricle with a fixative solution containing 4% paraformaldehyde. Subsequently, trigeminal ganglia were extracted, post-fixed for 1 h, followed by incubation in a sucrose solution (30%) for 24 h. Cryosections of 14 µm embedded in OCT were obtained, and sections were permeabilized with 1% Triton X-100, followed by blocking with 5% cold water fish jelly for 2 h. Immunostaining of the tissue was performed using a recombinant anti-TRPM8 antibody (ab109308, Abcam, Cambridge, MA, United States of America) and mouse anti-β-III-tubulin (sc-80016, Santa Cruz Biotechnology, Santa Cruz, CA, United States of America) (Cornejo et al., 2020; Hernández-Ortego et al., 2022). Secondary conjugated antibodies FITC and TRITC were acquired from Jackson Laboratory (1:1000 dilution). All antibodies were incubated in a blocking solution of 5% cold water fish jelly and 0.5% Triton X-100 at 4°C overnight and washed after incubation with PBS three times per 10 min each. Whole-mounted trigeminal samples (Fluoromount) were observed and analyzed in an Olympus FluoView FV1000 confocal microscope with a ×20 objective, Kalman 2, and 1–2x digital zoom. At least five longitudinal slices per ganglia (separated by at least 100 µm), focusing on the maxillary area, were imaged. All parameters, including laser intensity and detector gain, were consistent across all images acquired and conditions. Image processing was conducted using ImageJ software, employing a Gaussian filter and threshold adjustments for the manual counting of positive cells. A neuron was classified as positive if its raw fluorescence intensity was at least twice that of the background signal of a non-positive neuron. The number of cells positive for TRPM8 channels (TRPM8(+) or TRPM8-ir) over the total number of β-III-tubulin-positive neurons was quantified per slice and averaged across the sections.

### 2.10 Temperature stimulation

Coverslip pieces with plated neurons were placed in a microchamber and continuously perfused (∼1 mL/min) with solution warmed at ∼34°C. Bath temperature was adjusted with a water-cooled computer-controlled CS-1 Peltier device (Cool Solutions Research Devices, Carrigaline, Ireland), with the outlet close to the imaging field and controlled by a feedback device. Cold sensitivity was investigated with ∼40 s duration ramp-like temperature drops from 34ºC to 19°C, applied in the control solution and in the presence of different compounds applied using the same perfusion system.

### 2.11 Experimental protocols

The temperature threshold of IoN CSNs was first determined by Ca^2+^ imaging. The contribution of the brake potassium current I_KD_ to the thermal threshold of IoN CSNs was assessed by using 100 µM 4-AP as a blocker as in González et al. (2017). For patch-clamp experiments, trigeminal neurons were recorded under current- or voltage-clamp after determining the temperature threshold by Ca^2+^ imaging as in Madrid et al. (2006); 2009. In current-clamp mode, neurons were held at −60 mV by current injection, and a series of hyperpolarizing and depolarizing 500 ms current steps (Δi = 10–100 pA depending on input resistance) were delivered at a rate of 0.2 Hz to the cell. Using this protocol, we were able to determine resting membrane potential, input resistance, rheobase current, spike duration and shape, inward rectification index, and firing pattern. To measure the brake current I_KD_ in IoN CSNs under voltage-clamp conditions, cells were initially held at −50 mV. A 500 ms hyperpolarizing pulse to −120 mV was used to remove the inactivation of I_KD_ (Viana et al., 2002; Madrid et al., 2009) and to estimate the hyperpolarization-activated current I_h_ (Orio et al., 2009; 2012). The slow outward current measured 1 s after membrane potential was returned to −40 mV was taken as I_KD_ (Madrid et al., 2009; González et al., 2017). To determine the I_TRPM8_ current in IoN CSNs, cells were held at −50 mV and cooled from 34 to 20°C to measure the TRPM8-dependent cold-sensitive current (I_cold_); the difference between the current at both temperatures was considered as I_cold_, and the maximal TRPM8-dependent current was taken as the inward current potentiated by 1 µM WS-12 at 20°C (I_cold+ws-12_) as in (Rivera et al., 2021).

### 2.12 Data analysis

Data are reported as the mean ± SEM (standard error of the mean) as indicated. Unless mentioned otherwise, when comparing two mean values, statistical significance (*p* < 0.05) was assessed using Student’s unpaired, two-tailed *t*-test. For the unpaired *t*-test, Welch’s correction was applied in the case of unequal variances. For multiple comparisons of means, two-way ANOVA was performed in combination with Bonferroni’s *post hoc* test. Fisher’s (*F*) exact test was used to compare populations. Data analysis was performed using PRISM™ 5 (GraphPad Software, San Diego, CA, United States of America). All exact *p* values, statistical tests, and sample sizes are reported in the main text or figure legends.

### 2.13 Reagents and drugs

4-aminopyridine (4-AP, A78403) was purchased from Sigma-Aldrich (St. Louis, MO, United States of America). (1R,2S,5R)-N-(4-methoxyphenyl)-5-methyl-2-propan-2-ylcyclohexane-1-carboxamide (WS-12, 3040), Tetrodotoxin (TTx, 1078) was purchased from Tocris Bioscience (Minneapolis, MN, United States of America), and (S)-1-Phenylethyl (2-aminoethyl) (4-(benzyloxy)-3-methoxybenzyl) carbamate (PBMC, 10-1413) was purchased from Focus Biomolecules (Plymouth Meeting, PA, United States of America).

## 3 Results

### 3.1 Nocifensive behavior of injured mice in response to innocuous cold is linked to an increase in cold sensitivity of sensory neurons contributing to the infraorbital branch

**FIGURE 1:**
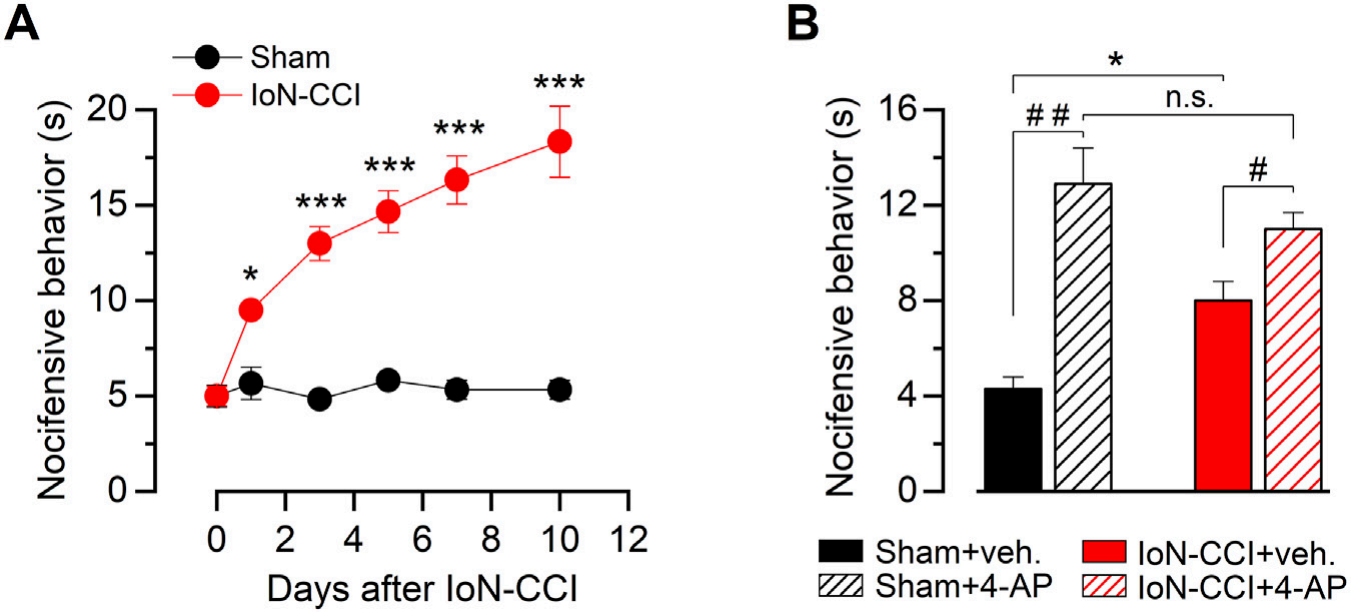
Nocifensive behavior in response to innocuous cold stimulation in sham and injured mice. **(A)** Time course of the cold-evoked nocifensive behavior assessed by acetone evaporative test in mice at the ipsilateral vibrissal pad, evaluated at zero (basal), one, three, five, seven, and 10 days after injury (see Methods). Red dots correspond to IoN-CCI animals, and black dots correspond to sham-operated mice; n = 6 animals for each group. **(B)** Bar graph summarizing nocifensive behavior after application of acetone to the ipsilateral vibrissal pad in a different set of sham and IoN-CCI animals, before and after pharmacological suppression of I_KD_ by local injection of 10 mM 4-AP or vehicle at day ten. Intergroup analyses of nociceptive behavior scores in A and B were performed by means of two-way ANOVA followed by the Bonferroni *post hoc* multiple-comparisons test: *p < 0.05; ***p < 0.001. Intragroup analyses in B were assessed by means of paired Student’s t-test (^##^ *p* = 0.0045, n = 7 sham animals; ^#^ *p* = 0.0382, n = 6 IoN-CCI mice).

Chronic constriction of the infraorbital branch induces nocifensive responses to innocuous cold stimulation on the vibrissal pad in region IoN-CCI mice compared to the sham-operated animals. Although we observed some differences in the allodynic response displayed by individuals after the same procedure, we consistently found that IoN-CCI induced a significant increase in the nocifensive behavior compared to their basal levels or sham-operated animals. Altered evaporative-cooling-evoked responses in the orofacial region appeared on the first day after surgery in the injured group, reaching a near-maximal value between the fifth and seventh day (Figure 1A). This sensory alteration remains stable for at least 1 month after nerve damage (see Figure 6D).

Since the pharmacological suppression of I_KD_ in nerve terminals of primary somatosensory neurons of intact animals induces cold-evoked nocifensive behaviors similar to those observed in response to cold in injured mice (Madrid et al., 2009), and the downregulation of the I_KD_ current has been proposed as a key molecular mechanism underlying cold allodynia triggered by peripheral nerve injury (González et al., 2017), we tested the effect of the I_KD_ blockage by 4-AP in the skin pad innervated by the IoN 10 days after surgery. In sham mice, this treatment produced enhanced acute nocifensive responses to acetone evaporation compared to the preinjection of vehicle (Figure 1B). In contrast, local acetone evaporation induces large nocifensive responses in IoN-CCI mice, and I_KD_ blockage potentiated these responses to a much lesser extent compared to sham-operated animals (Figure 1B). Thus, since IoN-CCI mice show a reduced sensitization by I_KD_ suppression, we hypothesize that orofacial cold allodynia could be, at least in part, the result of a reduction of the molecular target of this blocker in injured animals, *i.e.*, the Kv1 channels responsible for this critical brake current in primary sensory neurons.

### 3.2 IoN-CCI induces both an increase in the proportion of CSNs and a strong shift of the threshold in their cold-evoked responses to higher temperatures

**FIGURE 2:**
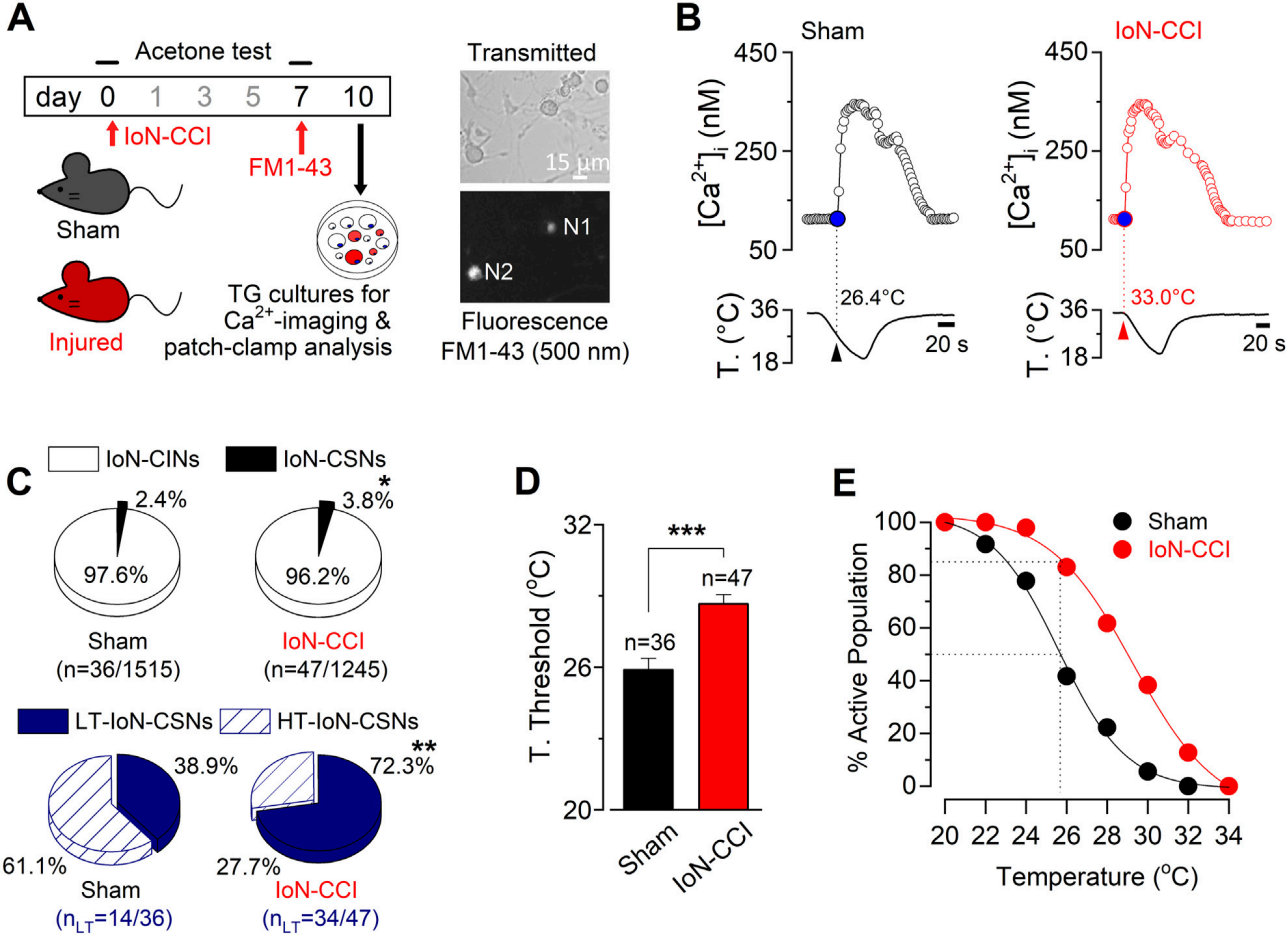
Increased cold sensitivity of trigeminal neurons contributing to the IoN in response to axonal damage. **(A)** Left panel: schematic representation highlighting the behavioral tests, FM1-43 retrograde labeling *in vivo*, and cultures of TG from sham and IoN-CCI mice. Right panels: transmitted (upper panel) and FM1-43 fluorescence (lower panel) images of IoN trigeminal neurons from a sham mouse in culture; N1 is a FM1-43 (+) CSN; N2 corresponds to a FM 1-43 (+) cold insensitive neuron (IoN-CIN). FM1-43 labeling *in vivo* was performed 3 days before culture. **(B)** Ratiometric [Ca^2+^]_i_ response to cold of representative FM1-43 (+) IoN CSNs from a sham (left panel) and an IoN-CCI mouse (right panel), recorded simultaneously with the temperature of the bath (lower traces). Arrowheads and dotted lines indicate the T^°^ threshold. **(C)** Pie plots showing the percentage of the different populations of CSNs and CIN from the infraorbital branch (FM1-43 (+)) in sham and IoN-CCI conditions. HT- and LT-correspond to high- and low-threshold IoN CSNs, respectively (*p = 0.0337, **p = 0.0034; Fisher’s *F* exact test). **(D)** Bar plot of temperature thresholds exhibited by neurons from sham (n = 36 CSNs) and IoN-CCI (n = 47 CSNs) mice. Cold thresholds were compared using a two-tailed unpaired Student’s t-test (***p < 0.001). Error bars are ±sem. **(E)** Percentage of active population recruited during a cooling ramp for IoN CSNs from sham and IoN-CCI animals.

As damage-triggered cold allodynia has been associated with changes in the proportion of CSNs (González et al., 2017), we explored the properties of cold-evoked responses in trigeminal neurons from both groups of animals. To compare the cold-sensitivity of trigeminal neurons from IoN-CCI and sham mice, we used intracellular Ca^2+^ imaging in dissociated TG neurons during rapid temperature reductions 10 days after surgery. Primary sensory neurons contributing to the infraorbital branch of the trigeminal nerve were identified by FM1-43 fluorescent retrograde labeling (Figure 2A) (see Methods), and the percentages of stained neurons observed in cultures from sham and IoN-CCI mice were alike. Similar to our previous findings in TG neurons (Madrid et al., 2009; Piña et al., 2019; Rivera et al., 2020; 2021), we have found that cold sensitivity among individual IoN neurons also varies in a wide range of temperatures. Figure 2B shows representative responses to cold stimulation of IoN CSNs from a sham and an IoN-CCI mouse. We found that the mean cold threshold of IoN CSNs from the sham group was 25.9 ± 0.5°C (n = 36) (ranging from 19.8 to 33.4°C), while the mean temperature threshold of cold-evoked responses of IoN CSNs from injured animals was 28.7°C ± 0.3°C (n = 47) (ranging from 23.7 to 33.5°C) (***p < 0.001, unpaired Student’s t-test).

Interestingly, although CSNs represent about 12% of total primary sensory neurons in TG (Viana et al., 2002; Madrid et al., 2006; Parra et al., 2010; Piña et al., 2019), only just above 2% of the neurons contributing to the IoN were sensitive to temperature drops (Figure 2C), suggesting a reduced basal cold-sensitivity among the fibers of this region compared to sensory neurons innervating other trigeminal territories. Notably, 10 days after surgery, the proportion of CSNs increases by almost 60% among the IoN neurons in cultures from injured mice compared to sham animals (2.4% (36/1515) vs. 3.8% (47/1245); *p = 0.0337, Fisher’s exact test) (Figure 2C). Among primary somatosensory neurons, cold thermoreceptors can be operationally classified as low-threshold (LT-) and high-threshold (HT-) CSNs (Thut et al., 2003; Madrid et al., 2009), where LT-CSNs are those neurons whose thermal threshold corresponds to temperatures above 26.5°C signaling pleasant cold, and the HT-CSNs are the neurons activated by temperatures below this value, signaling cold discomfort (Mauderli et al., 2003). In sham animals, we found that LT-IoN-CSNs represented 38.9% of the population of cold-sensitive primary sensory neurons of the maxillary branch (mean threshold = 28.8 ± 0.5°C, n = 14). The rest of them (61.1%) correspond to the HT-IoN-CSNs, with a mean temperature threshold of 24.1 ± 0.4°C (n = 22). In contrast, in IoN-CCI mice, 72.3% of CSNs contributing to the IoN correspond to low-threshold ones (mean threshold = 29.9 ± 0.3°C, n = 34), while HT-IoN-CSNs were less frequent (27.7%; mean threshold = 25.3 ± 0.2°C, n = 13) (Figure 2C, lower pie plots; **p = 0.0034, *F* test). On average, chronic constriction of the infraorbital branch induces a shift of the thermal threshold of ∼2.8°C to higher temperatures in injured animals compared to the sham group (Figure 2D; ***p < 0.001, unpaired Student’s t-test). The amplitude of cold-evoked responses of IoN CSNs of both groups was alike (*p* = 0.7890, unpaired Student’s t-test; data not shown). In Figure 2E, we represented the cumulative population of IoN CSNs activated by temperature reductions in these sets of experiments. Notice that at a temperature recruiting 50% of the IoN CSNs in sham-operated animals, over 85% of the CSNs are responding in the IoN-CCI group.

Altogether, these results suggest that the neural peripheral mechanisms underlying painful hypersensitivity to innocuous cold in injured animals involve an increase in the proportion of IoN CSNs in response to this form of axonal damage, along with a higher cold sensitivity of individual TG neurons contributing to the maxillary branch.

### 3.3 Reduced I_KD_ is associated with the shift of the thermal thresholds to higher temperatures exhibited by IoN CSNs from CCI animals

**FIGURE 3:**
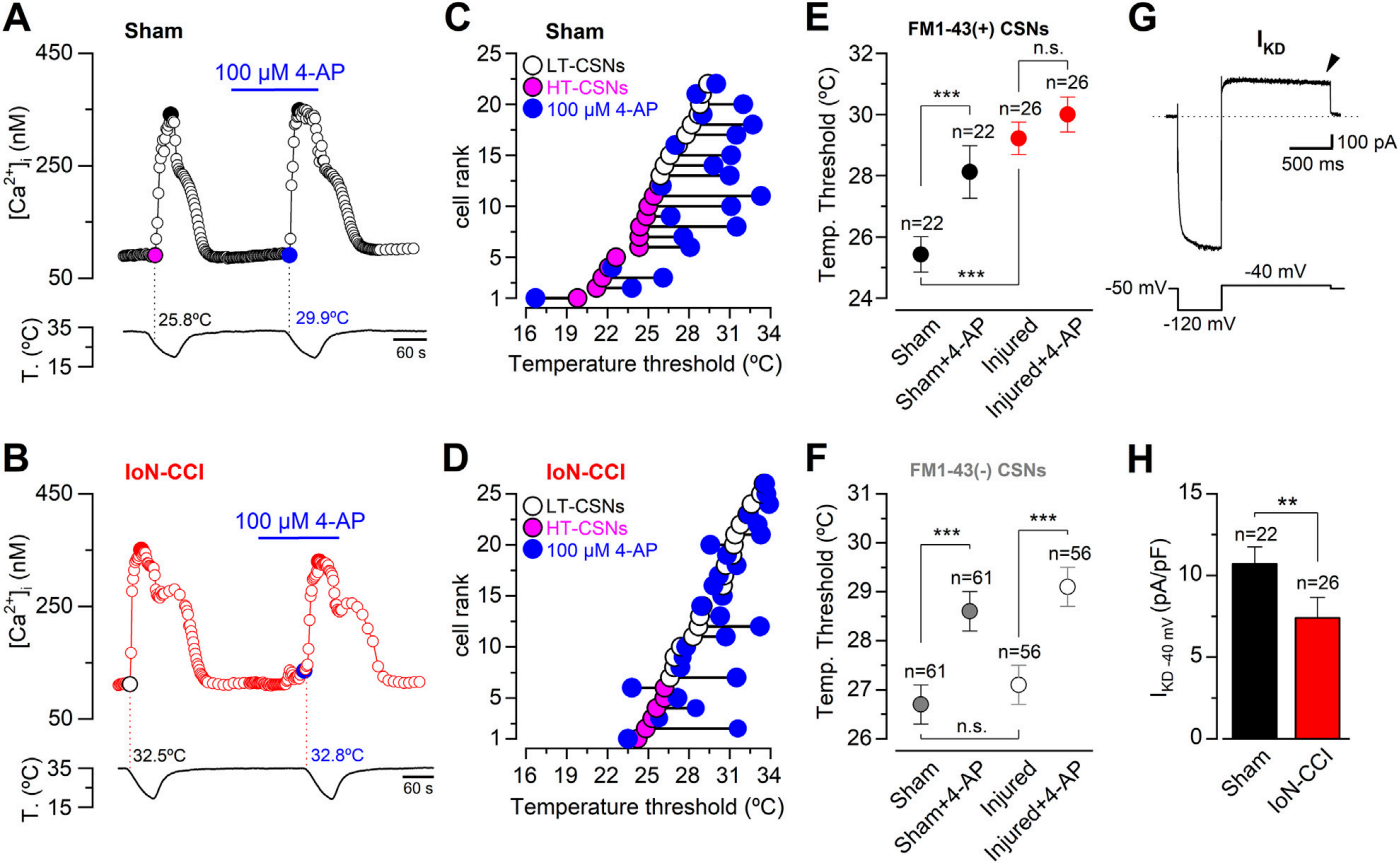
Effect of the pharmacological suppression of I_KD_ on thermal threshold of retrogradely labeled CSNs from sham and IoN-CCI mice. **(A)** Ratiometric [Ca^2+^]_i_ response in a representative IoN CSN from the sham group during two consecutive cooling stimuli in control solution and in the presence of 100 µM 4-AP. The cold threshold of this neuron was shifted to a higher temperature by 4-AP, from 25.8 to 29.9°C (black dotted lines). **(B)** Ratiometric [Ca^2+^]_i_ response in an IoN CSN from the IoN-CCI group during consecutive cooling ramps in control solution and in the presence of 100 µM 4-AP. In this neuron, the cold threshold was unaffected by the I_KD_ blocker (32.5 versus 32.8°C, red dotted lines). **(C)** Summary of the individual effect of 100 µM 4-AP on temperature threshold of cold-evoked responses in 22 IoN CSNs from the sham group. **(D)** Summary of the individual effect of 100 µM 4-AP on temperature threshold of cold-evoked responses in 26 IoN CSNs from IoN-CCI mice. **(E)** Mean values of the cold threshold of CSNs in sham and sham + 4-AP (black dots), and IoN-CCI and IoN-CCI in the presence of the I_KD_ inhibitor (red dots). Note that the mean thermal threshold of IoN-CCI CSNs was virtually unaffected by the pharmacological suppression of I_KD_. Neurons in A to E and G-H correspond to FM1-43 (+) CSNs contributing to the IoN. **(F)** Mean values of the cold threshold of FM1-43 (−) CSNs in sham and sham + 4-AP (gray dots), and IoN-CCI and IoN-CCI in the presence of the I_KD_ inhibitor (white dots). Neurons in F correspond to FM1-43 (−) CSNs recorded in the same fields of neurons from the infraorbital branch (FM1-43 (+)) in sham and IoN-CCI animals. Note that the mean threshold of IoN-CCI CSNs in the FM1-43 (−) group was equally sensitive to the pharmacological suppression of I_KD_ than the cold threshold of the IoN CSNs from sham mice. The significance of the temperature threshold shifts after treatment with 4-AP **(E, F)** were assessed using paired Student’s t-test [***p = 0.0002 and n. s. p= 0.0781 **(E)**; ***p = 0.0002 and ***p < 0.0001 **(F)**]. In E and F, temperature thresholds exhibited by CSNs from sham and IoN-CCI mice were compared using an unpaired Student’s t-test (***p < 0.0001 and n. s. p= 0.5172, respectively). **(G)** Whole-cell current (top trace) from a representative IoN CSNs from a sham animal during a bipolar voltage protocol (bottom trace). A hyperpolarizing pulse to −120 mV from a holding potential of −50 mV was applied before reaching the subthreshold membrane potential of −40 mV to reveal I_KD_ (see Methods). I_KD_ was measured at −40 mV one second after the return from the holding potential of −120 mV (black arrowhead) in the presence of 0.5 µM TTx. **(H)** Bar plot summarizing the mean I_KD_ current density in IoN CSNs from sham and IoN-CCI mice (Mann-Whitney test: **p = 0.0086). All error bars in this figure correspond to ±sem.

**FIGURE 4:**
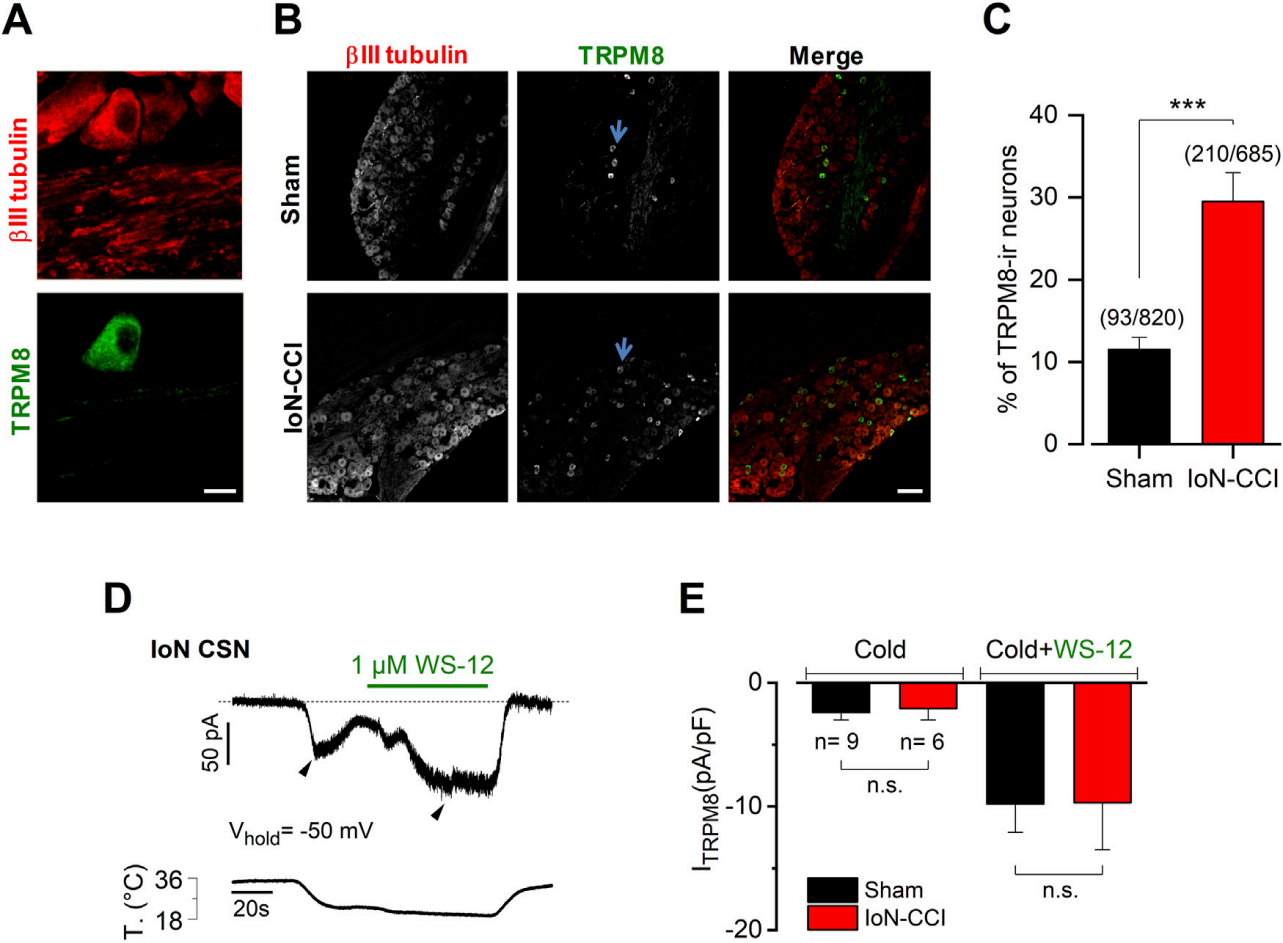
IoN-CCI in mice induces an increase in the proportion of TRPM8-expressing neurons without altering the mean I_TRPM8_ density **(A)**. Magnification of a region showing a TRPM8(+) neuron identified by immunostaining in the TG from a sham mouse. βIII tubulin is in red (1:1000); TRPM8 in green (1:100). Scale bar: 10 µm. **(B)** Representative slices of TG from sham and injured mice showing the increase of TRPM8(+) neurons after IoN injury. In the merge, βIII tubulin is in red and TRPM8 in green. Scale bar: 100 µm. Representative TRPM8(+) neurons are indicated by light blue arrows. **(C)** Bar plot summarizing the percentage of TRPM8(+) (TRPM8-ir) neurons identified in both groups (unpaired Student’s t-test with Welch’s correction; ***p = 0.0005) (n = 3 animals in each condition). **(D)** Simultaneous recording of membrane current (top trace) and bath temperature (bottom trace) during a sustained cooling step (20°C) combined with application of WS-12 (1 µM) in a representative IoN CSN from a sham animal (V_hold_ = −50 mV). The dotted line indicates the zero current level. **(E)** Bar plot summarizing the mean I_cold_ and I_cold+WS-12_ (I_TRPM8_) current density measured at the pick of both currents (black arrowheads in D) obtained in nine IoN CSNs from sham mice and six IoN CSNs from IoN-CCI animals; Mann-Whitney test: n.s. p= 0.7679 and 0.8329, respectively.

The temperature threshold of CSNs is tightly regulated by the brake current I_KD_ (Viana et al., 2002; Madrid et al., 2009). Thus, in primary afferents innervating different somatosensory territories, cold sensitivity is dampened by this inhibitory fast-activating slow-inactivating outward K^+^ current depending on Shaker-like Kv1.1 and Kv1.2 channels, acting as an excitability brake that sets the thermal threshold of both cold thermoreceptor neurons and a subset of nociceptive fibers (Viana et al., 2002; Madrid et al., 2009; González et al., 2017; Pertusa and Madrid, 2017). In this scenario, we wondered whether the alterations observed in the temperature threshold of IoN trigeminal neurons from injured animals could be explained by I_KD_ downregulation, as suggested by the acetone evaporative cooling assays conducted in 4-AP injected animals (see Figure 1B). Using intracellular Ca^2+^ imaging, we first explored the result of pharmacological suppression of I_KD_ on cold-evoked responses of trigeminal neurons from sham and IoN-CCI groups. To this end, we evaluated the effect of 100 µM 4-AP, a well-characterized and reversible I_KD_ blocker (Viana et al., 2002; Madrid et al., 2009; González et al., 2017; Piña et al., 2019), on the thermal responses of CSNs belonging to the maxillary branch. Figures 3A, B show the different results of I_KD_ inhibition on the cold-evoked responses of representative neurons from sham and IoN-CCI animals, respectively. In Figures 3C, D, the thermal threshold of IoN CSNs from sham and injured mice were plotted individually, according to the initial value, from highest to lowest, as HT- (magenta) and LT- (white) IoN CSNs. The temperature threshold of the cold-induced response of each neuron under 100 µM 4-AP was represented by blue circles. In neurons from sham animals, as has been reported on sensory neurons from trigeminal and dorsal root ganglia (Madrid et al., 2009; González et al., 2017; Piña et al., 2019), the temperature threshold of IoN CSNs was shifted to higher temperatures in 63.6% of the cells (14 of 22) in the presence of the I_KD_ blocker (Figure 3C). In one of the 22 CSNs depicted in Figures 3C, 4-AP shifted the thermal threshold of the neuron to a lower temperature, and seven showed no significant variation (ΔT< ±1°C). In this set of sham IoN CSNs, where the initial mean cold threshold was 25.4 ± 0.6°C (n = 22), the inhibition of the brake current shifted this value to 28.1 ± 0.9°C (***p = 0.0002, paired Student’s t-test) (Figure 3E). In contrast, in the 26 CSNs from the IoN-CCI group studied in the same conditions, the mean temperature threshold remained practically unaffected by 4-AP (29.2 ± 0.5 versus 29.9 ± 0.6°C; n. s. p= 0.0785, paired Student’s t-test) (Figures 3D, E). Accordingly, the thermal threshold of 15 of these IoN CSNs remained invariant despite the pharmacological suppression of the I_KD_, eight neurons showed a threshold shifted to higher temperatures, and three lowered the temperature threshold of their cold-evoked responses (Figure 3D).

In the same cultures, we also monitored the effect of 4-AP on neurons without FM1-43 fluorescent retrograde labeling, which most probably correspond to neurons innervating other trigeminal territories (Kuramoto et al., 2024). Remarkably, we found that the mean thermal threshold of unlabeled CSNs from sham and injured animals were alike and that the suppression of I_KD_ in these neurons shifted their mean cold thresholds to higher temperatures by more than 2°C in the presence of 4-AP in both groups (Figure 3F). These results suggest that the functional changes that explain the reduction of 4-AP sensitivity in IoN neurons from injured mice are confined mainly to the neurons contributing to the infraorbital branch in IoN-CCI animals.

IoN CSNs from injured mice show a mean cold threshold ∼2.8°C lower than neurons from sham animals, *i.e.*, shifted to higher temperatures (see Figure 2D). This variation was similar to the one observed after the pharmacological suppression of I_KD_ in IoN CSNs from sham mice (∼2.7°C; see Figure 3E). In addition, IoN CSNs from injured animals are less sensitive to 4-AP than neurons from sham mice, consistent with the idea that the brake I_KD_ current could be diminished in these neurons in response to nerve damage. To explore this possibility, we determined the mean current density of I_KD_ in IoN CSNs from both groups at −40 mV, a membrane potential subthreshold to the action potential firing in these neurons, where this brake potassium current exerts its functional role (Viana et al., 2002; Madrid et al., 2009). Figure 3G shows the recording of a representative I_KD_ in an IoN CSNs from a sham animal. We found that mean I_KD_ current density is reduced in IoN CSNs from injured mice compared to sham animals (10.7 ± 1.1 pA/pF, n = 22, in sham mice versus 7.2 ± 1.3 pA/pF, n = 26, in IoN CSNs from IoN-CCI animals; **p = 0.0086, Mann-Whitney test) (Figure 3H). This reduction of the I_KD_ could contribute to the increased cold sensitivity observed in IoN CSNs after nerve damage, explaining both the shift in thermal threshold and its lower sensitivity to 4-AP.

Altogether, these findings suggest that chronic peripheral nerve damage in the maxillary branch of the trigeminal nerve increases the orofacial cold sensitivity of IoN-CCI animals, at least in part, by reducing the functional expression of the 4-AP-sensitive molecular targets responsible for the I_KD_ current.

### 3.4 Injured mice exhibit an increase in the proportion of trigeminal TRPM8(+) neurons with no changes in the mean I_TRPM8_ density

The TRPM8 channel is the central molecular entity responsible for cold detection in primary sensory neurons under physiological conditions (for reviews, see (Babes et al., 2011; McCoy et al., 2011; Almaraz et al., 2014; Madrid and Pertusa, 2014; Pertusa et al., 2023)). TRPM8 also has a critical role in neuropathic cold pain since painful cold hypersensitivity is highly reduced in TRPM8 knockout animals (Colburn et al., 2007), and an increase in TRPM8 expression has been linked to the development and maintenance of cold allodynia in response to axonal damage (Xing et al., 2007; Su et al., 2011; 2017; Knowlton et al., 2013). We wondered if IoN damage also induces changes in the expression pattern of TRPM8 channels. Using immunostaining of the TG in both groups of animals, we found that the proportion of TRPM8-immunoreactive (ir) neurons is increased in the TG ipsilateral to the peripheral nerve damage (Figures 4A–C). TRPM8(+) neurons in TG sections from sham mice were ∼12% (11.5% ± 1.5%; n = 12 fields from three animals, 93 neurons from a total of 820 cells evaluated), consistent with the expression of this channel previously reported in this sensory ganglion (Viana et al., 2002; Madrid et al., 2006; Parra et al., 2010; Piña et al., 2019). In contrast, we found a rise in the percentage of TRPM8(+) neurons in the sections of TG obtained from IoN-CCI mice (29.5% ± 3.5%; n = 12 fields from three animals, 210 of 685 cells evaluated; ***p = 0.0005, unpaired Student’s t-test with Welch’s correction). In these experiments, the tissue samples were taken exclusively from the region of the trigeminal ganglia enriched in neurons corresponding to the infraorbital branch, and although we cannot assure that all these neurons are specifically innervating the injury site, these primary afferents likely belong to the damaged branch of the trigeminal nerve. These results suggest that the damage of the infraorbital branch also induces an increase in the expression of the TRPM8 channel in trigeminal neurons, which could be a part of the molecular mechanism leading to orofacial cold allodynia.

We also explored if IoN damage induces a variation of the TRPM8-dependent current that could contribute to the differences in temperature sensitivity observed among CSNs from sham and IoN-CCI animals. The direct determination of functional TRPM8 channels in IoN CSNs from both groups of mice was obtained using whole-cell voltage clamp recording at −50 mV, during cold stimulation (I_cold_) and a combination of cold plus 1 µM of the selective TRPM8 channel activator WS-12 to reach the maximal activation of TRPM8 channels (I_cold+WS-12_) (Rivera et al., 2021) (Figure 4D). We found that the mean I_TRPM8_ density in IoN CSNs from injured mice seems to be indistinguishable from the one obtained in sham animals’ neurons (Figure 4E).

Collectively, these results suggest that in addition to the brake current downregulation, IoN injury induces an increase in the proportion of TRPM8(+) trigeminal neurons without necessarily altering the mean TRPM8-dependent current in IoN CSNs.

### 3.5 The electrophysiological properties of IoN CSNs from sham and injured mice reveal the recruitment of formerly silent nociceptive neurons in response to axonal damage

**FIGURE 5:**
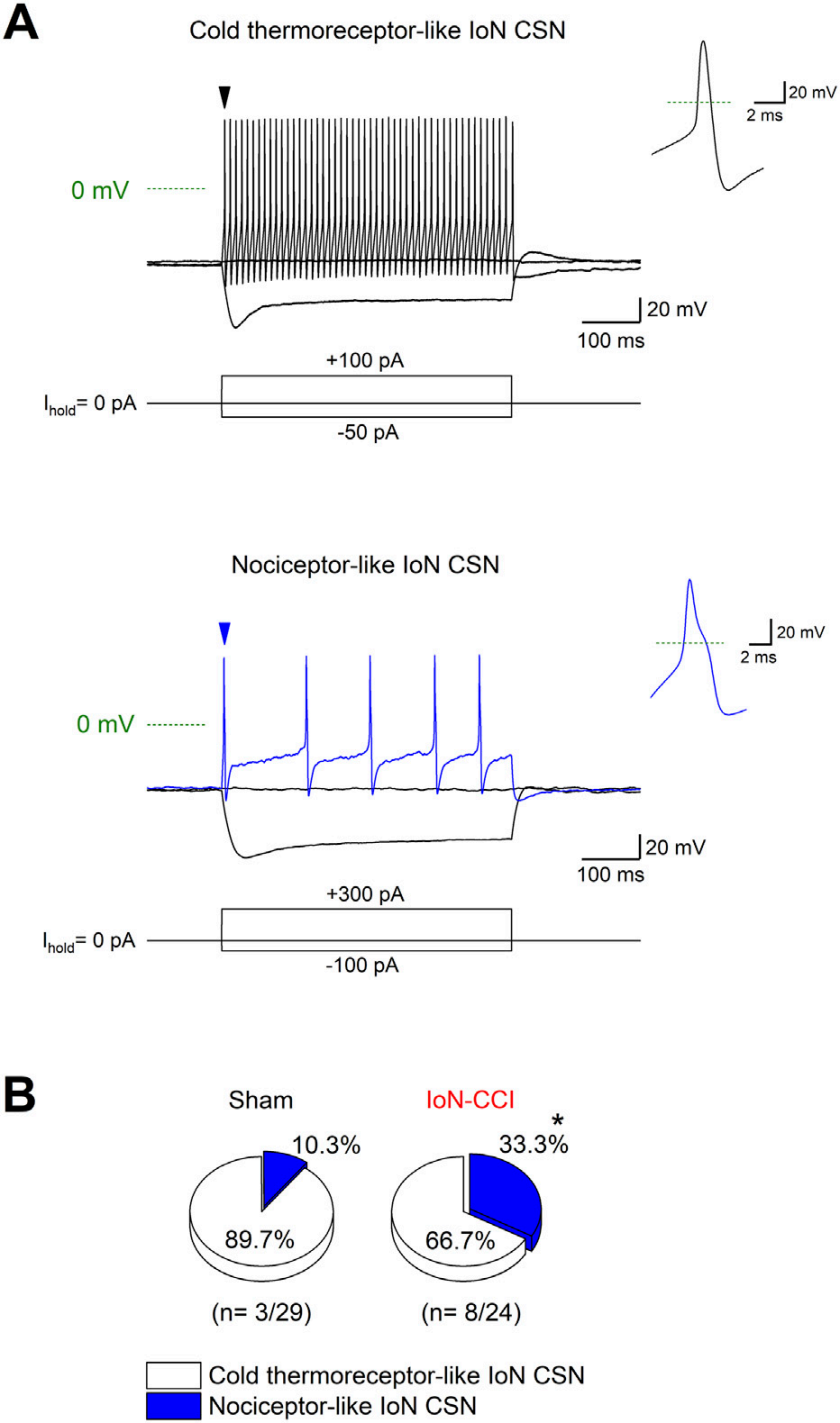
Electrophysiological properties of IoN CSNs. **(A)** Voltage responses to 500 ms hyperpolarizing and depolarizing current pulses (I_ext_) (bottom traces) from a representative cold thermoreceptor-like (upper panel) and a nociceptor-like (lower panel) CSNs. Both neurons correspond to IoN CSNs obtained from sham mice. Note the sag at negative membrane potentials. Insets at right: first action potential of each neuron (black and blue arrowheads in the upper and lower panels, respectively); note the inflection (hump) in the repolarizing phase of the action potential of the neuron in the lower panel. **(B)** Pie plots summarizing the percentage of IoN CSNs with cold thermoreceptor- and nociceptor-like phenotypes (*p = 0.0495; Fisher’s exact test) found in IoN CSNs from sham and injured mice.

The damage-induced TRPM8 expression in trigeminal neurons lacking this channel combined with the downregulation of I_KD_ could result in the recruitment of formerly cold-insensitive neurons into the innocuous range, explaining the increase observed in the proportion of IoN-CSNs in TG cultures after injury. These recruited neurons could correspond to cold thermoreceptors with very low sensitivity to cold temperatures or to nociceptive fibers. Considering that canonical cold-thermoreceptors commonly fire trains of narrow action potentials in response to depolarizing current injections (Reid et al., 2002; Viana et al., 2002; Madrid et al., 2009), and polymodal nociceptor neurons fire wide action potentials, often with an inflection (or hump) in the falling phase (∼2 ms at the half-amplitude) (Fang et al., 2005; González et al., 2017), we explored the neural phenotype of IoN CSNs studying the passive and active membrane properties of neurons from both groups. Using hyperpolarizing and depolarizing current pulses under current-clamp conditions, the resting membrane potential, input resistance, rheobase current, inward rectification index, action potential shape, and firing properties were determined (Figure 5A; Table 1). In cultured neurons from IoN-CCI mice, we found that 33.3% of IoN CSNs showed a hump in the repolarizing phase, compared to the 10.3% observed in neurons from sham animals (Figure 5B), suggesting the recruitment of a population of nociceptor-like neurons signaling cold-evoked pain in response to mild cold stimulation in injured mice. The rest of the parameters were alike in both groups.

**TABLE 1:** Active and passive membrane properties of retrogradely labeled CSNs from the infraorbital branch from sham and injured mice.

	Cold sensitive IoN neurons	Resting potential (mV)	Input resistance (MΩ)	Rheobase current (pA)	AP duration (ms)	Inward rectification index (%)	Firing freq. at 2x rheobase (Hz)
Sham (n = 29)	Thermoreceptor-like (n = 26)	−50.0 ± 1.7	321 ± 55	249 ± 54	0.75 ± 0.06	48.2 ± 2.8	24 ± 5
	Nociceptor-like (n = 3)	−44.1 ± 5.3	435 ± 78	175 ± 18	1.30 ± 0.20*	28.5 ± 6.0	18 ± 14
IoN-CCI (n = 24)	Thermoreceptor-like (n = 16)	−47.4 ± 2.2	278 ± 75	266 ± 72	0.65 ± 0.08	43.1 ± 4.7	23 ± 4
	Nociceptor-like (n = 8)	−45.8 ± 2.3	432 ± 138	191 ± 51	1.28 ± 0.15**	30.3 ± 6.4	33 ± 7

Cold sensitivity of the IoN neurons was previously determined using Ca^2+^ imaging. Resting membrane potential was measured at I_hold_ = 0 pA. Input resistance was determined by measuring the voltage drop induced by a hyperpolarizing current step reaching −120 mV from a holding current of 0 pA. Rheobase current was measured as the minimal current required to trigger an action potential by depolarizing current steps. AP, duration was measured at half the amplitude of the first action potential evoked by a depolarizing current pulse (*p = 0.0287 for sham and **p = 0.0030 for IoN-CCI). The inward rectification index was calculated as 100x (V_peak-_V_steady-state_)/V_peak_ during the voltage drop induced by a hyperpolarizing current pulse of 500 ms reaching a peak voltage of around −120 mV. The firing frequency in each IoN CSN, was calculated from the number of spikes at 2x rheobase current counted in a 500 ms period. Mann-Whitney test in all the cases.

Thus, our results support the idea that the functional unbalance of TRPM8 and the brake current I_KD_ in response to IoN damage shifts the thermal threshold of IoN cold thermoreceptors and induces cold sensitivity in formerly silent neurons of the maxillary branch most probably connected to nociceptive routes, contributing to the allodynic phenotype in IoN-CCI animals.

### 3.6 Exploring two strategies to revert the nociceptive phenotype of injured mice

**FIGURE 6:**
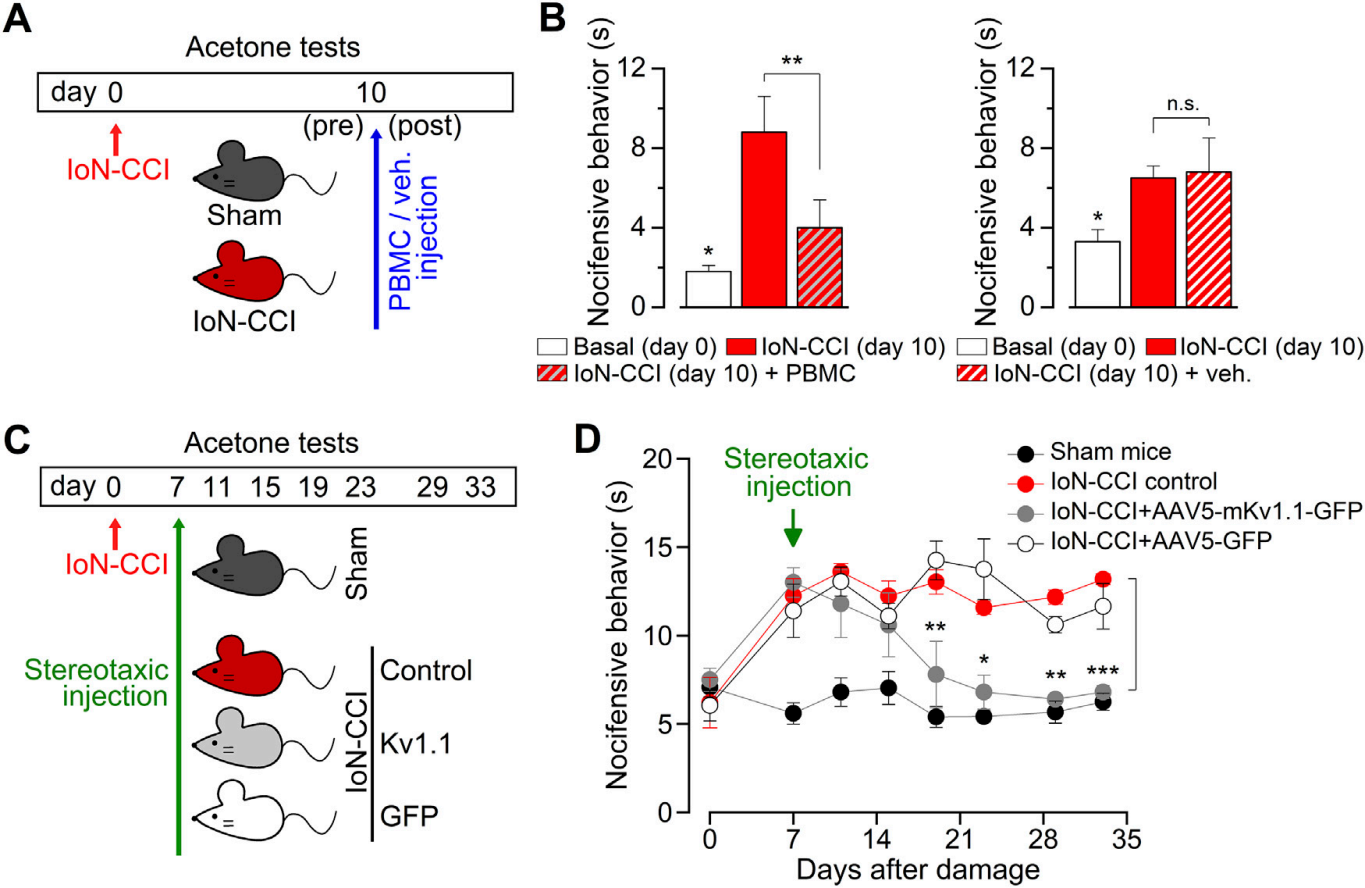
Reversion of the nociceptive phenotype of IoN-CCI mice by the pharmacological suppression of TRPM8 channels and by the AVV5-based expression of Kv1.1 channels. **(A)** Schematic representation of the time-line, including peripheral nerve injury, behavioral tests, and PBMC or vehicle injection in sham and IoN-CCI mice. **(B)** Bar graphs summarizing the duration of nocifensive behavior after application of acetone to the ipsilateral vibrissal pad in IoN-CCI animals before and after pharmacological suppression of TRPM8 by systemic application of 10 mg/kg of the TRPM8 channel blocker PBMC (left panel) (n = 4 mice) or vehicle (right panel) (n = 4 mice). Statistical significance was assessed by paired Student’s t-test (**p = 0.0080 and n. s. p= 0.7449, respectively). Basal responses at day 0 were significantly different to the allodynic responses at day ten in both groups (*p = 0.0416 and *p* = 0.0244, respectively; paired Student’s t-test). **(C)** Scheme of the time-line, including peripheral nerve injury, behavioral tests, and stereotaxic injections in the different groups of animals. Cold-evoked nocifensive behavior was assessed by evaporative cooling test (see Methods) and evaluated at 0 (basal), 7 (previous to the stereotaxic injection), 11, 15, 19, 23, 29, and 33 days after injury. **(D)** Dot plot showing the reversion of the nocifensive response to cold in the vibrissal pad by expression of mKv1.1 channel in the TG using stereotaxic injection of AAV5-mKv1.1-GFP, AAV5-GFP, and vehicle (1 μL, 10^13^ particles; 0.25 μL/min; coordinates 4.3 mm rostral, 1.5 mm lateral and 6.24 mm ventral to the lambda) in injured mice that develop painful hypersensitivity to innocuous cold (n = 5 to 6 animals in each condition). Statistical significance between IoN-CCI (control) animals and IoN-CCI + AAV5-mKv1.1-GFP was assessed by a two-way ANOVA followed by the Bonferroni *post hoc* test (*p < 0.05, **p < 0.01, ***p < 0.001 for control vs. AAV5-mKv1.1-GFP) (n.s. for control vs. AAV5-GFP).

The increase in the population of TRPM8-expressing neurons observed in injured animals could alter the dependent sensory input at the IoN, exacerbating orofacial cold-induced responses. In this scenario, we explored the possibility that the pharmacological blockage of the TRPM8 channel could reduce the cold-evoked nociceptive responses of IoN-CCI animals. We administrated intraperitoneal 10 mg/kg of PBMC, a specific and effective TRPM8 channel blocker (Knowlton et al., 2011), examining the nociceptive responses using the evaporative cooling assay before and 40 min after the injection of the antagonist in a group of injured animals with orofacial cold allodynia at day ten after surgery (Figure 6A). We found that PBMC significantly reduced the nocifensive response to acetone stimulation in the vibrissal pad (Figure 6B), reinforcing the idea that TRPM8 channels are necessary for the painful response of IoN-CCI animals and that the treatment with this blocker may be considered an effective pharmacological tool to revert cold allodynia in this model.

Additionally, considering the reduction of the brake current in response to axonal damage, another approach to revert painful cold hypersensitivity could be upregulating the functional expression of the Kv1 channels responsible for I_KD_ (Madrid et al., 2009; Fan et al., 2014). With this in mind, we expressed one of the molecular counterparts of the I_KD_, the Kv1.1 channel, using adeno-associated viral serotype 5 (AAV5) vectors that efficiently transduce primary somatosensory neurons (Guedon et al., 2015). To test its efficacy, we first explored the effect of AAV5-driven expression of Kv1.1 in cultured trigeminal neurons as an initial approach. We found that AAV5 vectors effectively transduce over 60% of TG neurons in culture and that the expressed Kv1.1 channels reduce the cold sensitivity of these neurons, shifting the thermal threshold of their cold-evoked responses by more than 2°C to lower temperatures (Supplementary Figure S1), suggesting that this strategy could remediate the increase in the cold sensitivity induced by IoN-CCI *in vivo*. Therefore, we expressed this channel in TG using AAV5-Kv1.1 delivered by stereotaxic injection, and we explored the effect of this maneuver on orofacial cold allodynia (Figure 6C). We found that the group of injured animals treated with AAV5-Kv1.1, but not with AAV5-GFP, reverted the cold-evoked nociceptive behavior from day 12 after stereotaxic injection of viral particles, a condition that remains stable at least until day 33 after nerve damage (Figure 6D). These results support the idea that the upregulation of even one of the brake’s Kv1 channels may also be effectively used to reduce painful cold hypersensitivity in this form of peripheral nerve damage at orofacial territories.

**FIGURE 7:**
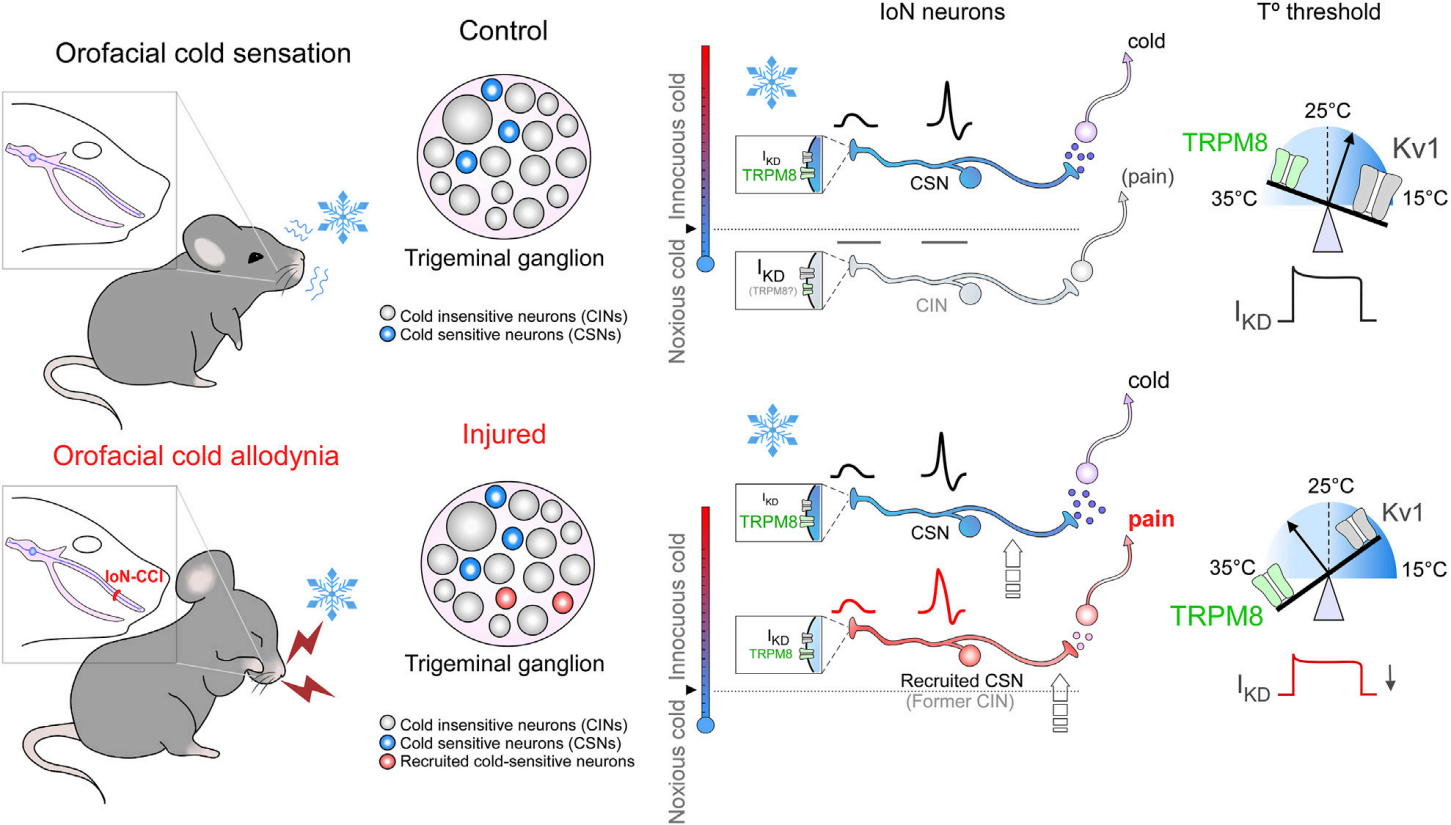
Schematic model of thermal sensitivity of IoN primary sensory neurons in sham and injured conditions. Upper panels: schematic representation of cold detection mechanisms in IoN primary sensory neurons under physiological conditions. Boxes in nerve endings depict the ion channels involved in detecting cold stimuli at the different subtypes of primary afferents (CSNs and CINs). The relative temperature ranges in which these nerve endings would be excited by cold are shown by the colored thermometers at the left of the boxes; activation of these neurons lead ultimately to distinct cold and cold-evoked pain sensations. The CSN represents the (canonical) LT- and HT- IoN CSNs, signaling pleasant cold and cold discomfort depending on their thermal thresholds. The CIN represents recruitable nociceptor-like neurons that become cold-sensitive into the non-noxious range after peripheral nerve damage. The size of labels reflects the relative functional expression of TRPM8 and Kv1 channels in the different subclasses of primary afferents. Scales on the peripheral nerve endings at right depict the functional counterbalance of the excitatory (TRPM8, green) and brake (Kv1.1-1.2, gray) channels (I_KD_) involved in the molecular tuning of cold detection. Lower panels: the schematic representation of these mechanisms in injured (IoN-CCI) mice emphasizes the functional variations in response to peripheral nerve damage. The relative change in the size of the labels reflects the expected changes in functional expression levels for the different ion channels post-peripheral nerve injury. After axonal damage, the reduction in the functional expression of I_KD_ would recruit nociceptive neurons usually activated by noxious cold temperatures inducing pain, which will respond to mild cold temperatures in IoN-CCI mice, and will shift the thermal threshold of IoN CSNs to higher temperatures (arrows indicate these transitions). Thus, after nerve injury, the reduction in the functional expression of I_KD_ and an increase in TRPM8-expression will increase the cold sensitivity of HT-IoN-CSNs signaling cold discomfort and will recruit silent nociceptive neurons normally activated by very low temperatures signaling cold-evoked pain.

A schematic representation of our results regarding the thermal sensitivity of trigeminal neurons of the IoN before and after peripheral nerve injury is shown in Figure 7.

## 4 Discussion

We have found that after damaging the infraorbital branch of the trigeminal nerve, an increase in the proportion of IoN CSNs and a shift in their thermal thresholds to higher temperatures contribute to the orofacial cold allodynia observed in injured animals. This exacerbated sensitivity to cold emerges due to a reduction in the Kv1.1-1.2-dependent brake potassium current I_KD_ accompanied by a rise in the TRPM8-expressing population of trigeminal neurons. However, we do not rule out any additional functional variation occurring in other ion channels than those we explored in this study. The contribution of the excitatory TRPM8-dependent cold responses and the Kv1-dependent I_KD_ current shown here has been also observed in different models of cold hypersensitivity induced by peripheral nerve injury, although the functional unbalance of these two counterparts differs depending on the territory and the form of nerve damage evaluated. For instance, in trigeminal sensory fibers innervating the eye surface, the peripheral injury by deep surgical ablation of trigeminal corneal nerves mainly induces an enhanced functional upregulation of TRPM8 channels (Piña et al., 2019). In that study, the increase in the TRPM8-dependent excitatory input in damaged corneal CSNs produces a rise in these fibers’ basal firing and menthol-evoked responses, sensibly increasing the basal tearing rate in injured animals. Significantly, the brake current was not altered in response to that form of peripheral nerve damage of corneal fibers. Thus, in that model, a disturbance mainly dependent on the increase in the functional expression of TRPM8 was sufficient to induce higher cold sensitivity of the trigeminal neurons from injured animals, by recruiting formerly cold-insensitive neurons with a functional phenotype corresponding to canonical cold thermoreceptor fibers (Piña et al., 2019). In contrast, painful hypersensitivity to innocuous cold induced by CCI of the sciatic nerve in mice has been suggested to be mainly due to the downregulation of the I_KD_ current (González et al., 2017; Pertusa and Madrid, 2017). Remarkably, although no major differences in TRPM8 function were found in that study, the cold sensitivity of sensory neurons from control and injured animals strongly depends on TRPM8 since pharmacological suppression of this channel by PBMC abrogates the cold-evoked responses of CSNs from both groups. Interestingly, in this spinal somatosensory territory, the functional unbalance due to a decrease in the I_KD_-dependent inhibitory component also causes a shift in their cold temperature threshold of 2°C to warmer temperatures, with an increase in the proportion of CSNs due to the recruitment of formerly cold-insensitive nociceptive neurons (González et al., 2017; Pertusa and Madrid, 2017). This different territory- and injury-specific modulation of these two functionally antagonistic molecular counterparts underlying cold sensitivity could explain the dissimilar observations regarding the involvement of TRPM8 in cold allodynia after nerve injury. Thus, while this sensory alteration has been linked to an increased expression of TRPM8 channels in response to axonal damage (Xing et al., 2007; Su et al., 2011; 2017; Knowlton et al., 2013; Piña et al., 2019) and cold allodynia is reduced in TRPM8 knockout mice reinforcing the significant role of this channel (Colburn et al., 2007), it has also been reported that exacerbated painful cold sensitivity in response to peripheral nerve injury could not necessarily be correlated with significant variations in TRPM8 expression levels (Katsura et al., 2006; Namer et al., 2008; Caspani et al., 2009; González et al., 2017).

The alterations in the excitability of trigeminal neurons we observed here in response to IoN-CCI are confined to neurons contributing to the maxillary branch. These changes increase the cold sensitivity of a group of neurons that appears to show proportionally less cold-responding fibers than other somatosensory territories, given that just above 2% of the cultured IoN neurons are cold-sensitive into the innocuous range of low temperatures in basal conditions (see Figure 2C). Besides, since approximately 10% of the ipsilateral trigeminal neurons were retrogradely labeled by the fluorescent marker in sham and IoN-CCI mice, the characterization of the 2%–3% of that subpopulation of neurons was challenging. It has been reported that FM1-43 can label primary afferents depending on the activity of mechanosensitive channels, partially affecting mechano-activated currents (Drew and Wood, 2007; Villarino et al., 2023). This could contribute to lower efficacy in staining cold-sensitive fibers with low expression of these channels. Nevertheless, the same marker used in the ophthalmic branch revealed the expected proportion of 12% of CSNs among FM1-43-labeled neurons in intact animals (Piña et al., 2019), indicating that this tracer was able to stain different types of trigeminal afferents, including cold-thermoreceptors. Therefore, the low proportion of CSNs observed in the IoN in our study could reflect a trait of this trigeminal region compared to other somatosensory territories.

Given that the unbalance of TRPM8 and the brake channels occurs in a way that both changes move the equilibrium towards increased cold responsiveness, the functional effect induced by the damage of the infraorbital branch results in a robust shift in the thermal threshold (∼2.8°C in IoN CSNs) and the recruitment of sensory fibers that contribute to the nociceptive phenotype. Our conductances-based model of cold-sensitive neurons (González et al., 2017), predicts that a reduction in the I_KD_/I_TRPM8_ ratio is enough to allow a silent cold nociceptor-like neuron to fire into the cold-thermoreceptor temperature range and also transform a high-threshold cold thermoreceptor into a low-threshold CSN, explaining painful hypersensitivity to innocuous low temperatures. Moreover, this model also predicts that the thermal threshold is more sensitive to variations in I_KD_ than in I_TRPM8_, and that even a low density of a TRPM8-like current is enough to generate cold-evoked responses in primary afferents expressing low levels of Kv1 channels underlying the I_KD_. Since IoN-CCI increased the number of TRPM8-expressing neurons in the TG ipsilateral to the nerve damage, a combined effect also involving the downregulation of Kv1 channels should increase the chances of the appearance of cold-sensitivity in orofacial trigeminal fibers, in coincidence with our experimental findings.

Reinforcing the idea that downregulation of Kv1 channels is behind cold allodynia in several pathologies, MacDonald and colleagues, using *in vivo* Ca^2+^ imaging in mice, showed evidence that altered painful cold sensitivity observed in oxaliplatin-induced neuropathy, partial sciatic nerve ligation, and ciguatera poisoning models are related to unmasking silent cold-sensing neurons as a result of Kv1 channels functional reduction in nociceptive neurons expressing Nav1.8 and CGRPα (MacDonald et al., 2021). Thus, shifting thermal thresholds and recruiting formerly cold-insensitive sensory neurons that become cold-sensitive after injury emerge as a common functional feature of damage-triggered cold hypersensitivity, that could explain this sensory alteration in different models of peripheral nerve damage. Corroborating the relevance of the molecular counterparts of the I_KD_ in pain neurophysiology, it has been shown that Kv1.1 acts as a molecular brake in mechanical and pain sensitivity (Hao et al., 2013), and that the expression of Kv1.1 and Kv1.2 is decreased in neuromas of myelinated axons after injury (Calvo et al., 2016). Interestingly, peripheral nerve damage induces the downregulation of Kv1.2 mRNA and protein in injured dorsal root ganglia neurons through the damage-induced promotion of a long non-coding antisense RNA for Kv1.2 expression (Zhao et al., 2013; 2017; Fan et al., 2014; Li et al., 2015). An additional suggested mechanism behind the reduction of protein levels of this ion channel is the epigenetic silencing of the Kcna2 gene, due to an increase in the histone-lysine N-methyltransferase 2 activity (Liang et al., 2016) and the subsequent methylation of the Kcna2 promoter region (Zhao et al., 2017). Altogether, these results open an exciting possibility of an unbalance involving cold-sensitive excitatory channels and the functional downregulation of the Kv1-dependent brake current as a general mechanism to explain and tackle this sensory alteration in multiple peripheral neuropathies. Whether a similar molecular and neural mechanism contributes to the altered thermal sensitivity in other painful neuropathies, such as post-herpetic neuralgia or diabetic neuropathy, must be further explored.

In this scenario, different approaches that could effectively contribute to treating cold allodynia are the pharmacological inhibition of the excitatory component and/or the potentiation of the inhibitory brake current. Several TRPM8 blockers have been developed over the last years, although some of them present side effects, including alterations in thermoregulation and lack of potency and specificity (Izquierdo et al., 2021). Among these blockers, a promising example for this task is PBMC. Systemic application of this TRPM8 channel blocker has been previously shown to be effective in reducing cold hypersensitivity in the CFA-induced model of inflammatory pain and also in the sciatic nerve-CCI model of neuropathic pain (Knowlton et al., 2011). Nevertheless, its side effect on thermoregulation at high concentrations and the lack of effectivity attenuating cold-evoked pain reported in oxaliplatin-induced neuropathy are limitations that must be considered (Knowlton et al., 2011).

On the other hand, one possibility to enhance the I_KD_ current is to express Kv1.1 or Kv1.2 channels using viral-based delivery, and adeno-associated viral vectors stand out in this regard. AAV vectors remain episomal, persisting in a non-dividing state for years, an important advantage for effective and long-term modifications. Although we did not directly measure the potentiated currents in neurons from transduced mice, in our hands, AAV5-Kv1.1 efficiently transduce trigeminal neurons, allowing the expression of this channel and resulting in a substantial reduction of the cold-evoked nociceptive phenotype in injured animals. Moreover, AAV-based treatments have also been tested as a potential therapeutic tool for neuropathic pain, targeting different molecular entities that are relevant in pain neurophysiology (Xu et al., 2003; Gu et al., 2005; Hirai et al., 2014; Kim et al., 2020; Ji et al., 2023), including the manipulation of Kv1 channels (Fan et al., 2014; Guedon et al., 2015). Importantly, restoring of Kv1.2 expression using AAVs in damaged dorsal root ganglia diminished painful responses in allodynic animals (Fan et al., 2014). Thus, the results presented here and previous evidence suggest that Kv1.1-1.2 potassium channels represent promising molecular targets in the treatment of painful cold hypersensitivity in neuropathic pain, and that viral-based delivery designed to increase the functional expression of potassium channels underlying I_KD_ may be a potential strategy to revert damage-triggered cold allodynia, not only in orofacial neuropathic pain but also in other sensory alterations where these channels could also have a critical role.

Although our results suggest that both pharmacological suppression of TRPM8 using PBMC and AAV5-Kv1.1 transduction resulted in a substantial reduction of the cold-evoked nociceptive phenotype in injured animals, further studies are necessary to establish the most successful therapeutic approach to revert exacerbated cold sensitivity in different somatosensory territories and forms of peripheral nerve damage, especially to explore the possibility of combinatorial and long-term molecular manipulations of these key ion channels to effectively treat this distressing sensory alteration avoiding undesirable effects.

## 5 Conclusion

This study unveils the central role of TRPM8 and Kv1 channels in damage-triggered cold allodynia in orofacial neuropathic pain. TRPM8 channels and the potassium channels underlying the brake current I_KD_ are functionally unbalanced in trigeminal neurons in response to the damage of the infraorbital nerve. This unbalance enhances the cold sensitivity of high-threshold cold thermoreceptor neurons signaling cold-evoked discomfort and transforms a subpopulation of silent neurons signaling pain into neurons activated by mild cold temperatures. Our results provide a molecular and neural model for this form of pathological cold-evoked pain in orofacial territories in response to chronic peripheral nerve injury.

## Data Availability

The raw data supporting the conclusions of this article will be made available by the authors, without undue reservation.

## Abbreviations

CCI, Constriction Injury; IoN, Infraorbital nerve; IoN-CCI, Chronic Constriction Injury of the Infraorbital Nerve; AAV, Adeno-Associated Viral vectors; CSNs, Cold Sensitive Neurons; TG, Trigeminal Ganglia; 4-AP, 4-Aminopyridine (potassium channel blocker); PBMC, (S)-1-Phenylethyl (2-aminoethyl) (4-(benzyloxy)-3-methoxybenzyl) carbamate (TRPM8 channel blocker); Kv1.1, Voltage-gated Potassium channel family 1, member 1; Kv1.2, Voltage-gated Potassium channel family 1, member 2; TRPM8, Transient Receptor Potential Melastatin 8 channel; I_KD_, Kv1.1-1.2-dependent brake potassium current; WS-12, (1R,2S,5R)-N-(4-methoxyphenyl)-5-methyl-2-propan-2-ylcyclohexane-1-carboxamide (TRPM8 channel activator).
