# Supplementary material for: A functional unbalance of TRPM8 and Kv1 channels underlies orofacial cold allodynia induced by peripheral nerve damage
Source: Front Pharmacol. 2024 Dec 5;15:1484387. doi: 10.3389/fphar.2024.1484387 (PMC11655194; doi:10.3389/fphar.2024.1484387)
Supplement: Supplementary file 1 [file Image1.PDF]

Supplementary Material

Supplementary Figure

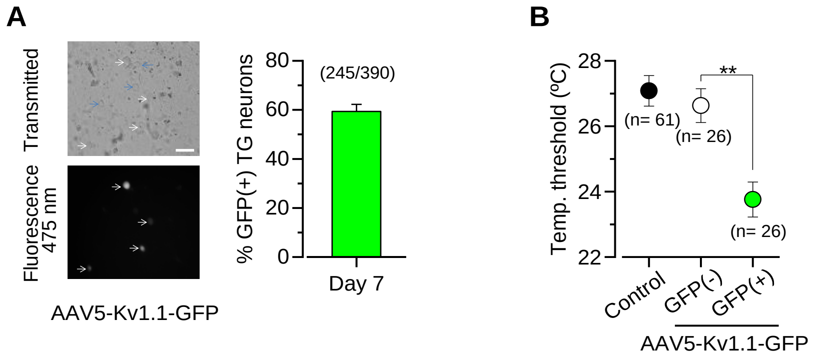

Supplementary Figure 1. AAV-based expression of Kv1.1 channels reduces the thermal sensitivity of cultured TG neurons. A. Transmitted (upper panel) and GFP fluorescence (lower panel) images in a field of AAV5-Kv1.1-GFP-transduced trigeminal neurons in culture. White arrows highlight GFP(+) neurons; light blue arrows correspond to GFP(-) neurons. The mean percentage of GFP-expressing neurons on day seven after transduction (89 fields from four cultures) is shown in the bar graph on the right panel (245 of 390 of neurons). **B.** Dot plot summarizing the thermal threshold of cold-evoked responses of cultured CSNs from trigeminal ganglia. In control is shown the mean thermal threshold of CSNs from non-transduced cultures (black dot). In cultures exposed to the AAVs, AAV5-mKv1.1-GFP(+) correspond to neurons expressing GFP (green dot) or not (AAV5-Kv1.1-GFP(-), white dot) (**$p < 0.001$, unpaired Student's *t* test). Note that Kv1.1-(GFP(+)) expressing neurons have a cold threshold ~3ºC shifted to lower temperatures compared to CSNs from non-transduced (GFP(-)) or to control (non-treated) cultures.
